# Single-stage total knee arthroplasty and femoral osteotomy for osteoarthritis with severe supracondylar deformity

**DOI:** 10.1186/s13018-021-02293-w

**Published:** 2021-02-20

**Authors:** Jing-yang Sun, Guo-qiang Zhang, Jun-min Shen, Yin-qiao Du, Tie-jian Li, Zong-jie Geng, Yong-gang Zhou, Yan Wang

**Affiliations:** 1 Medical School of Chinese PLA, Beijing, 100853 China; 2 Department of Orthopedics, The First Medical Center, Chinese People’s Liberation Army General Hospital, Fuxing Road, Haidian District, Beijing, 100853 China

**Keywords:** Extra-articular deformity, Total knee arthroplasty, Single-stage, Osteotomy, Long cemented stem

## Abstract

**Background:**

Knee osteoarthritis associated with extra-articular deformity (EAD) can confront the arthroplasty surgeons with challenges of bone resection and soft tissue balancing. The aim of this study was to describe a single-stage procedure associating corrective osteotomy with total knee arthroplasty (TKA), and to determine the outcome at mid- to long-term follow-up.

**Methods:**

A total of seven patients (seven knees) with knee osteoarthritis and supracondylar deformity were included in this study. Six patients were female, and one was male, with the median age of 62 years (range, 37-76 years). All patients were treated with single-stage TKA and femoral osteotomy. Osteotomy was fixed with long cemented stem. Hospital of Special Surgery (HSS) scores, collateral ligament laxity, and range of motion (ROM) were clinically evaluated preoperatively and at each follow-up. Radiographic parameters including the mechanical axis deviation (MAD), mechanical lateral distal femoral angle (mLDFA), mechanical proximal tibial angle (mMPTA), and joint line congruence angle (JLCA) were also measured. The occurrence of perioperative complications was recorded.

**Results:**

The median follow-up time was 91 months (range, 38-104 months). At the last follow-up, all components were stable and no patients required revision. Nonunion of the osteotomy occurred in one patient. In all patients, the lower limb mechanical alignment improved greatly. The mean angle of MAD was restored from 10.49±6.05 cm preoperatively to 1.11±4.97 cm postoperatively. The 90° mLDFA was almost acquired in all cases, with the postoperative value of 90.79±2.40°. After operation, the mMPTA improved from 84.18±6.13° to 91.33±3.13°. The JLCA changed from 2.94±1.61° to −0.71±3.50°. The median HSS score improved from 45 (range, 34-56) preoperatively to 90 (range, 82-97) postoperatively, with the outcome of all patients rated good to excellent. The median ROM improved from 70° (range 0–110°) preoperatively to 105° (range 90–125°) postoperatively. No instability of knee joint was observed. Complications included an intraoperative split fracture of distal femur and one case of wound exudation resulting from fat liquefaction.

**Conclusions:**

For knee osteoarthritis with femoral supracondylar deformity, single-stage TKA and corrective osteotomy was feasible but technically demanding. The use of long cemented stem for osteotomy fixation can provide reliable rotational control of the bone segments.

## Background

Component alignment determines functional outcome and survival in total knee arthroplasty (TKA). Specially, it is a technically demanding procedure to restore the physiological mechanical axis of lower limb in the context of severe extra-articular deformity (EAD). EADs are rare, mainly occurring secondary to fracture malunion, previous corrective surgery, congenital diseases, and bony metabolic diseases. Advanced osteoarthritis associated with these deformities can confront the arthroplasty surgeons with challenges of bone resection and soft tissue balancing [1, 2].

Correction of EAD can be achieved by TKA with intra-articular correction or combined with extra-articular corrective osteotomy [1–4]. While making choice between the two strategies, the magnitude, direction (varus, valgus, antecurvatum, recurvatum, and rotation), location (femur or tibia) of the deformity, and its distance from the knee should be taken into consideration [2]. EADs closer to the knee effect more on the mechanical axis [5]. Based on literatures, intra-articular bone resection and soft tissue release may not work well when EAD presents >= 10 degrees in the coronal plane or >= 20 degrees in the sagittal plane [5, 6]. Regarding EAD on femur, an extensive release can balance the knee in extension but will, possibly, cause laxity on the released side in flexion. This will lead to instability or compromise on the rotational mal-positioning of the femoral component. Sometimes, aiming for restoration of the normal mechanical axis, the planned resection may violate the collateral ligament origin [2, 7, 8]. Besides, severe sagittal or rotational deformity is difficult to compensate by the position of the component. Therefore, under those complex circumstances, corrective osteotomy to normalize long-bone anatomy is prudent.

Corrective osteotomy and TKA can be performed either simultaneous or successive. Compared with two-stage procedure, a single-stage TKA and corrective osteotomy is an attractive option, which is associated with shorter rehabilitation period, reduced risks by avoiding a second anesthesia application and operation, and lower spending [2, 9]. However, the single-stage procedure is technically challenging. Considering the extent of surgical exposure and length of operation, patients are prone to complications such as infection, nonunion, and pseudarthrosis formation of the osteotomy site [8, 10, 11]. Therefore, the single-stage procedure calls for careful preoperative planning and experience of both TKA and corrective osteotomy.

The technique of single-stage procedure is still scarcely described in literatures with limited number of patients. To date, various options for fixation of corrective osteotomy have been recommended, including plate, intramedullary nail, k-wire, and long press-fit stem [2, 8, 10, 11]. To our knowledge, few studies reported the use of long cemented stem for osteotomy fixation. In this study, single-stage procedure was performed in seven patients with advanced degenerative osteoarthritis of the knee accompanying with EAD on femur. All osteotomy was performed prior to bone cuts on femur and fixed with a long cemented stem. The purpose of this study was to determine the outcome at mid- to long-term follow-up and to provide an overview of our techniques.

## Patients and methods

The study was approved by our Institution Review Board. Between September 2011 and March 2017, a total of seven patients (seven knees) treated with single-stage TKA and femoral osteotomy were included in this study. No active infection and quadriceps weakness were present. No hardware was retained. Six patients were female, and one was male, with the median age of 62 years (range 37-76). The supracondylar EAD was related to femoral fracture mal-union in 1 patient, constitutional in 3 patients, and secondary to femoral osteotomy with over-correction in 3 patients. All knees had advanced osteoarthritis of grade 4 according to Kellgren–Lawrence criteria [12]. All patients preferred the one-stage procedure and written informed consent was obtained.

Hospital of Special Surgery (HSS) scores, collateral ligament laxity, and range of motion (ROM) were clinically evaluated preoperatively and at each follow-up. Based on HSS scores, clinical outcome was categorized as follows: excellent (>=85), good (70–84), fair (55–69), and poor (<55). Collateral ligament laxity was examined using lateral stress test. ROM of knee joint was measured using a goniometer and recorded as an approximate value with the interval of 5°, including the passive maximum flexion and flexion contracture.

A complete set of knee radiographs including anteroposterior views, lateral views, and full-length lower limb standing films were conventionally obtained preoperatively and postoperatively. Before surgery, if a rotational deformity was possibly present in the axial plane, we would turn to computerized tomography and three-dimensional reconstruction. The lateral radiograph revealed the antecurvatum or recurvatum deformity. The full-length radiograph revealed the varus or valgus deviation from the mechanical axis on coronal plane. From the full-length radiograph, the mechanical axis deviation (MAD), mechanical lateral distal femoral angle (mLDFA), mechanical proximal tibial angle (mMPTA), and joint line congruence angle (JLCA) were measured. During templating, we aimed to acquire a normal mechanical axis with the mLDFA valued 90° and without anteroposterior angulation. A wedge osteotomy was planned on the apex of the deformity according to the Paley’s principles of deformity correction [13].

### Surgical techniques

**Fig. 1:**
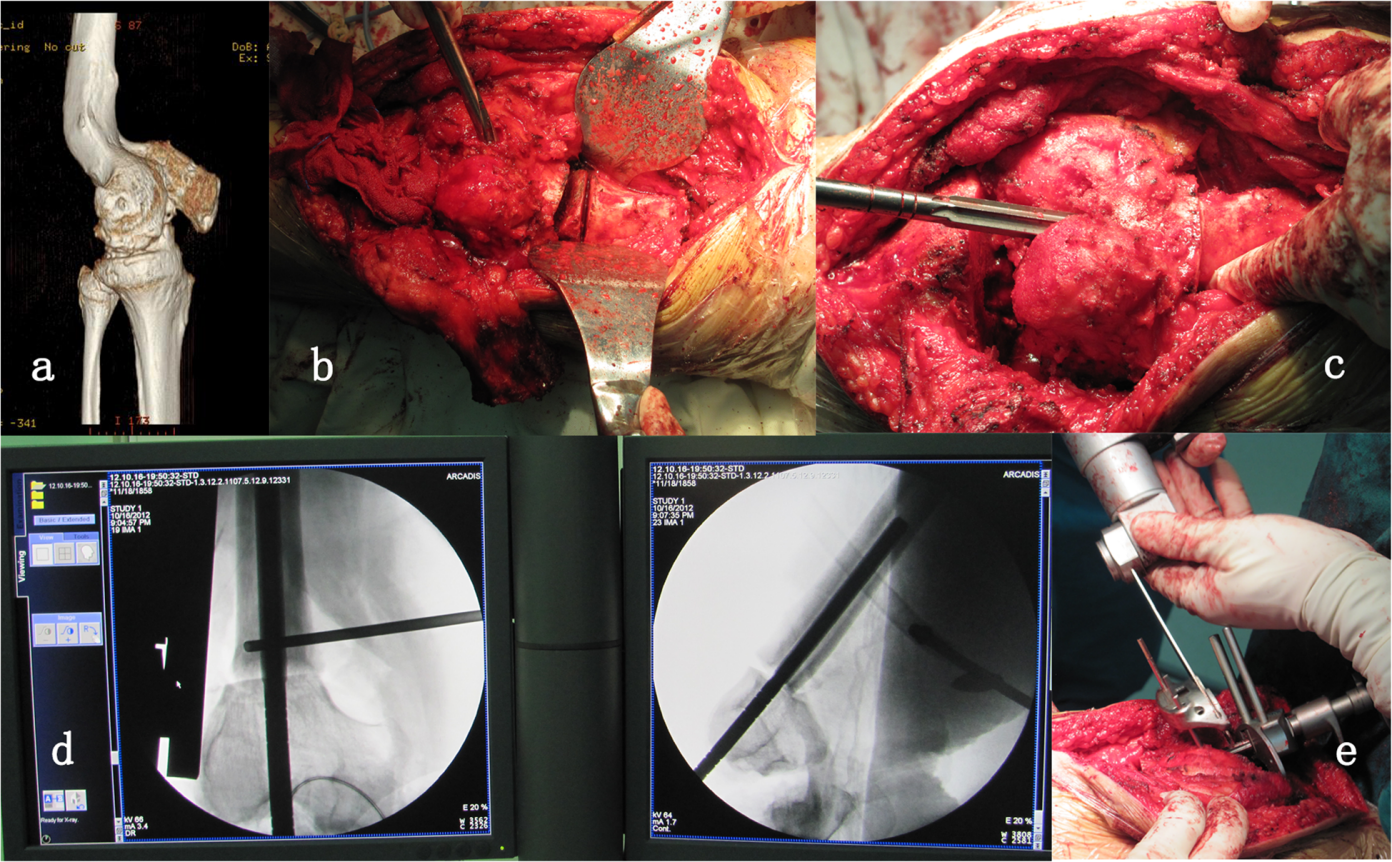
The procedure of one-stage TKA and femoral osteotomy. **a** Recurvatum deformity on femur. **b** A wedge osteotomy was performed at the center of the deformity. **c** With the separate bone segments stringed together and correctly aligned, the femoral medullary canal was reamed until the cortex of isthmus was engaged. **d** Adequate correction was confirmed with fluoroscopy. **e** Bridging the osteotomy site with an intramedullary alignment rod, standard bone cut at distal femur was performed

Single-stage TKA and femoral osteotomy with long cemented stem augmentation was applied in all patients. All surgeries were performed by one senior surgeon using a midline or the prior longitudinal incision under general anesthesia. A medial parapatellar arthrotomy was used and extended proximally to gain access to the distal femoral deformity. After bone resection of the tibia, the femoral medullary canal was opened through the center of distal femur and along its distal anatomical axis. The position of femoral osteotomy was identified under direct vision. The rotational alignment should be clearly marked with the electrocautery before osteotomy. No rotational deformity was required to be restored to the anatomical position. The asymmetrical flexion gap caused by abnormal rotation of distal femur was planned to be compensated by the rotational adjustment of femoral component with the use of gap balancing technique. An oscillating saw was used to perform a wedge osteotomy at the center of the deformity. Again, with the separate bone segments stringed together and correctly aligned, we reamed the femoral medullary canal using a rigid reamer until the cortex of isthmus was engaged. With the reamer in situ, adequate correction was confirmed with fluoroscopy. The osteotomy site was subsequently provisionally bridged with an intramedullary alignment rod. Standard femoral bone cuts were performed with a 5° or 7° valgus angle (Fig. 1). The bone defect and soft tissue balancing were then addressed. The long extension stem which could reach the femoral canal isthmus was used in all patients. During implantation of the femoral component, the distal part of stem was cemented to reliably stabilize the osteotomy. No bone graft was used for osteotomy site.

Posterior stabilized component was used in 6 patients (PFC Sigma, DePuy, Warsaw, IN). Constrained condylar knee was used in 1 patient (TC3, DePuy, Warsaw, IN). Among the 6 PFC components, fixed bearing was used in 5, and rotating platform in 1. Two patellae were resurfaced. Extension stem on tibial side was used in only 1 case. Quadriceps snip was applied in 1 case due to ankylosing knee. Bone defect of tibia in 1 case was treated by the technique of screws and cement. Patients’ demographic data and extra-articular deformities, and surgical information were summarized in Table 1.

**Table 1:** Demographic data, extra-articular deformity, and surgical information of the patients

Case	Gender	Age (years)	BMI (kg/m^2^)	Cause of deformity	Deformity (type/degrees)	Surgical time	Tourniquet (time/interval)	Type of implant	Extension stem (length, mm)	Tibial insert (type/thickness, mm)
1	M	37	28.39	Fracture malunion	Recurvatum 36.02	4° 40′	1° 39′ (1′) 28′ 1° 12 (2′)	CCK	175	CCK 10
2	F	76	31.25	Genu valgum	Valgus 11.70	2° 40′	1° 30′	PS	175	Plus 10
3	F	62	30.30	Previous osteotomy; over-correction	Varus 17.98 Antecurvatum 39.41	3° 15′	1° 12′ (1′) 16′ 51′ (2′)	PS	125	Conventional 10
4	F	74	27.83	Previous osteotomy; over-correction	Valgus 16.13 Antecurvatum 34.11	3° 25′	1° 18′	PS	175	Conventional 15
5	F	68	26.70	Genu valgum	Valgus 12.76	2° 50′	1° 22′	PS	125	Conventional 12.5
6	F	60	20.32	Previous osteotomy; over-correction	Varus 17.08	3° 32′	1° 10′ (1′) 20′ 57′ (2′)	PS	175	Plus 12.5
7	F	61	21.94	Genu valgum	Valgus 16.34	2° 58′	1° 37′	PS	175	Conventional 12.5

*BMI* body mass index, *F* female, *M* male, *CCK* constrained condylar knee, *PS* posterior stabilized

At the first day after surgery, patients were allowed full weight bearing, joint mobilization, and ambulation with a walker. All patients received postoperative intravenous antibiotic and deep venous thrombosis prophylaxis. Patients were asked for follow-up visit in regular intervals at 3 months, 6 months, and yearly after surgery. The occurrence of perioperative complications including nonunion of the osteotomy, fractures, thromboembolism, and infection was recorded.

### Statistical analysis

Failure of the operation was defined as revision of any component. Descriptive statistics, mean±standard deviation, or median and range were used to represent the demographics of the patients, radiographic parameters, ROM, and clinical scores. Paired *t* test was used to determine the difference between pre- and postoperative quantitative data. A *P* value < 0.05 denoted statistical significance. All data analysis was performed on SPSS 21.0 software (IBM Inc., Armonk, New York).

## Results

**Fig. 2:**
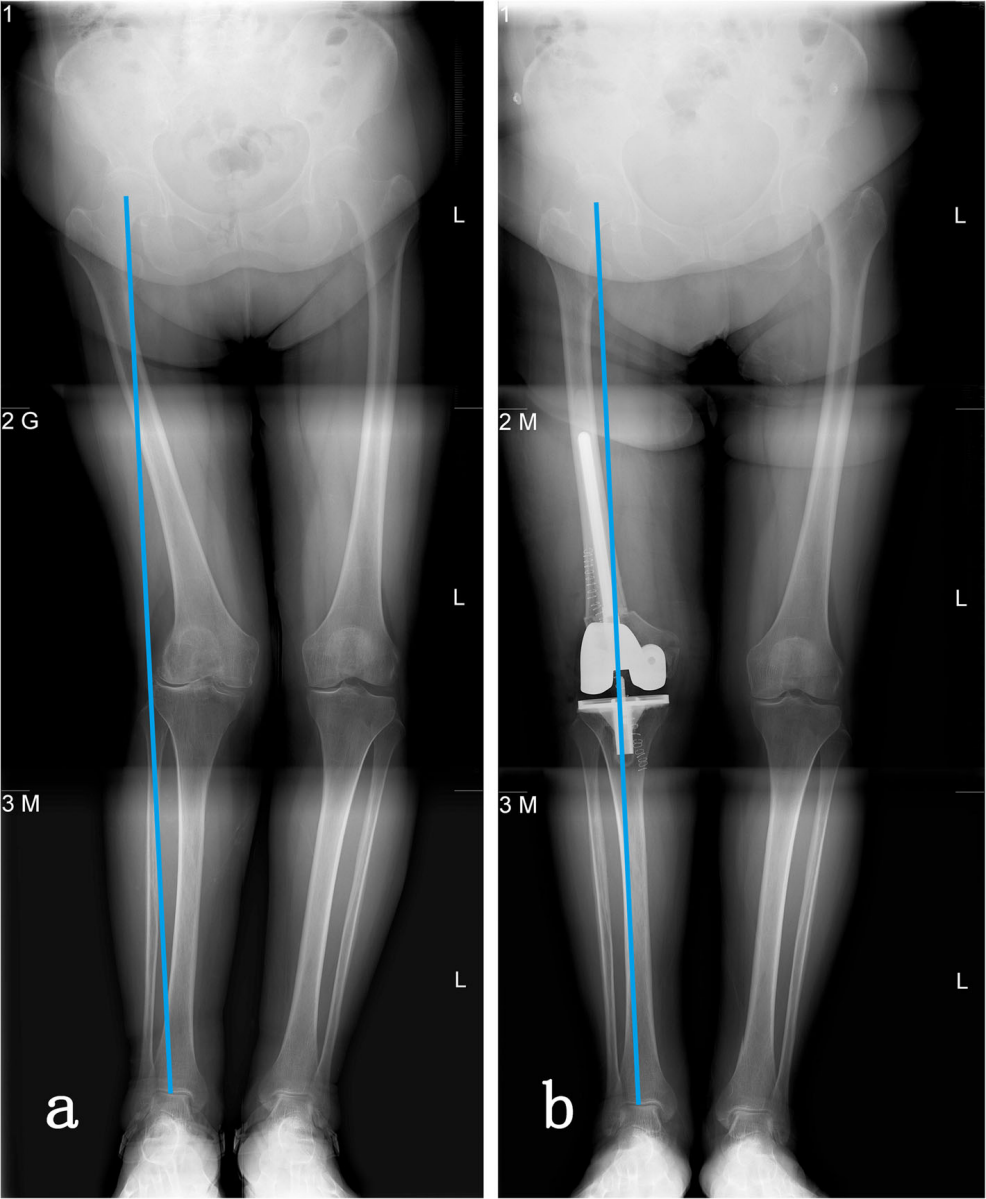
A 76-year-old women (case 2) with severely constitutional genu valgum of the femur was treated with one-stage TKA and femoral osteotomy. **a** Preoperative lower limb mechanical axis. **b** Postoperative lower limb mechanical axis

The median follow-up time for all patients was 91 months (range, 38-104 months). At the last follow-up, all components were stable and no patients required revision. At 6 months after operation, the osteotomy site healed in 6/7 cases. Nonunion of the osteotomy occurred in one patient, but without pain and any discomfort. In all patients, the lower limb mechanical alignment improved greatly. A representative case (case 2) with severely constitutional genu valgum of the femur was shown in Fig. 2. In this case, the mechanical axis was restored from the extreme outside to the midline of knee joint.

Clinical scores and ROM before and after total knee arthroplasty were shown in Table 2. The median HSS score improved from 45 (range, 34-56) preoperatively to 90 (range, 82-97) postoperatively, with the outcome of all patients rated good to excellent. No patients required the walking aid. No instability of knee joint was observed. Median preoperative ROM was 70° (range 0–110°) with a median flexion of 90° (range 0–110°) and a median flexion contracture of 10° (range 0–30°). At the last follow-up, median ROM was 105° (range 90–125°) with a median flexion of 110° (range 90–125°) and a median flexion contracture of 0° (range 0-5°).

**Table 2:** Clinical scores and ROM before and after total knee arthroplasty

Case	Follow-up duration (months)	HSS score		Maximum flexion		Flexion contracture	
		Preop	Postop	Preop	Postop	Preop	Postop
1	91	45	87	0	90	0	0
2	94	56	93	110	125	0	0
3	104	42	83	90	100	20	0
4	38	50	82	90	110	30	5
5	92	34	90	75	100	15	0
6	72	50	92	90	115	10	0
7	62	43	97	90	120	10	0

*ROM* range of motion, *HSS* hospital of special surgery

Regarding the limb alignment, the mean angle of MAD was restored from 10.49±6.05 cm preoperatively to 1.11±4.97 cm postoperatively. The 90° mLDFA was almost acquired in all cases, with the postoperative value of 90.79±2.40°. After operation, the mMPTA improved from 84.18±6.13° to 91.33±3.13°. The JLCA changed from 2.94±1.61° to −0.71±3.50°.

**Fig. 3:**
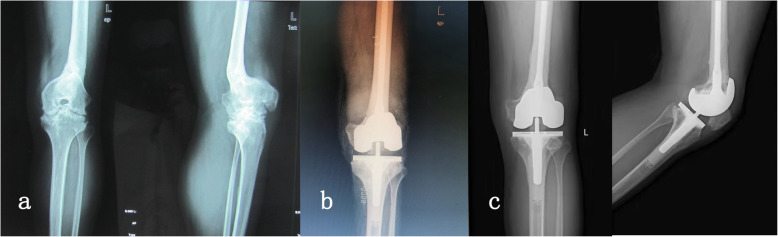
A 37-year-old man (case 1) with recurvatum deformity on femur was treated with one-stage TKA and femoral osteotomy using a constrained condylar knee. **a** Anteroposterior view and lateral view before surgery. **b** X-ray at the first day after surgery showing an asymmetrical gap. **c** Long-term X-ray (91 months postoperatively) showing a stable component and no sign of pronounced wear on tibial insert

**Fig. 4:**
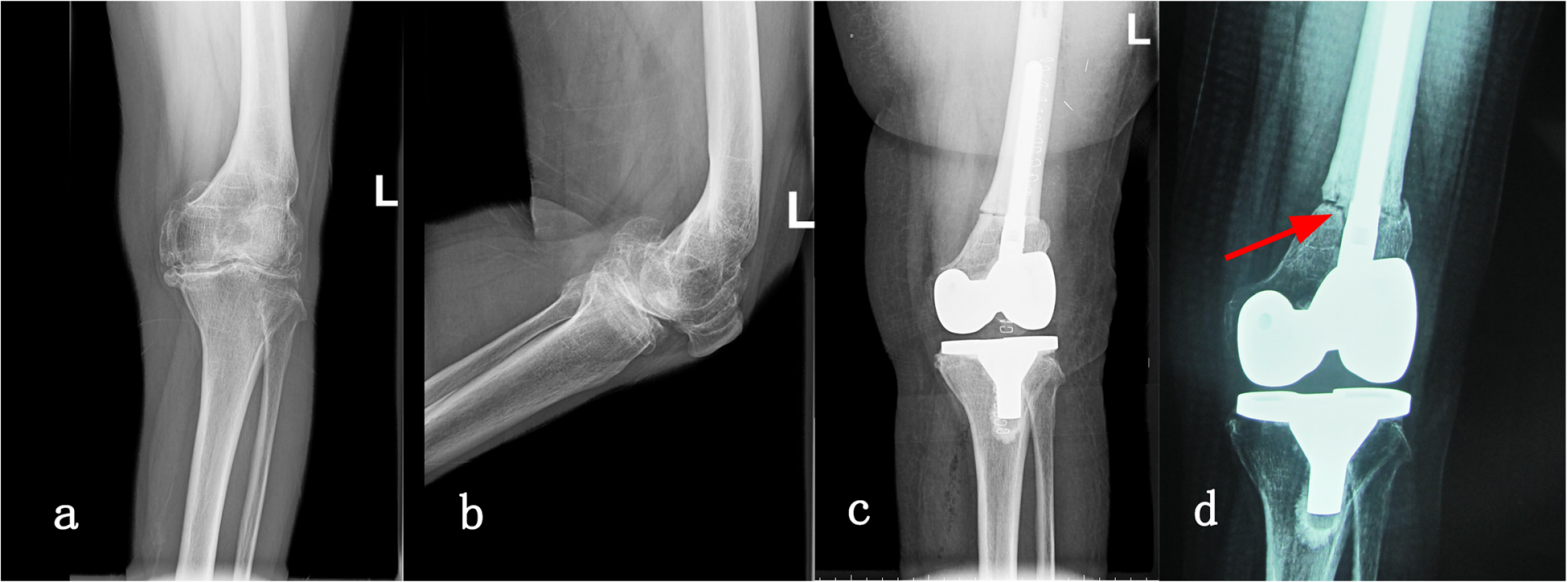
A 62-year-old women (case 3) with extra-articular deformity due to previous over-correction. **a**, **b** Anteroposterior view and lateral view before surgery. **c** X-ray at the first day after surgery. **d** Nonunion of the osteotomy resulting from the sandwiched cement

Symmetrical gap could not be gained in one knee (case 1) due to its complex three-dimensional deformities, and was finally compensated by a constrained condylar knee. To our relief, the latest radiograph showed a stable component and no sign of pronounced wear on tibial insert at the follow-up of 91 months postoperatively (Fig. 3). Intraoperative complication included a split fracture of distal femur in one patient during implanting the femoral component, which was fixed with two cortical screws and healed uneventful (case 5). Postoperatively, one patient had wound exudation resulting from fat liquefaction and underwent a debridement (case 3). In this case, the osteotomy developed nonunion. Sandwiched cement can be observed at the osteotomy site (Fig. 4). However, without evident symptoms, this patient denied any further intervention for the nonunion.

## Discussion

When TKA is indicated, the EAD must be considered to achieve satisfactory ligament balance, acceptable component alignment, and better restoration of the mechanical axis both in the coronal and sagittal planes [1]. A corrective osteotomy may be required if the intra-articular resection can jeopardize the collateral ligament insertion. Besides, it is also recommended when either the level of the bone cuts or the angle between them is unacceptable or uncorrectable due to severe or multiplanar extra-articular deformity [2].

Corrective osteotomy can be performed either as a separate procedure before the TKA or during the same stage as the TKA. Single-stage procedure seems more cost-effective due to the shorter treatment time, lower costs, and fewer operations. However, this combined procedure was associated with a great deal of increased blood loss, infection, and thromboembolism risks [8, 10, 11]. In our study, only 1 case developed wound exudation because of fat liquefaction and no event of thromboembolism occurred. Moreover, Deschamps et al. observed that among cases associating osteotomy with TKA, the gain in the functional score seemed less than in the group of isolated TKAs [14]. But in their series, the cases undergoing single-stage procedure appeared more severe and complex compared with the control group. Through the single-stage procedure, the clinical score of all our cases was rated good to excellent, which indicated its feasibility.

Several published studies have addressed combined TKA and femoral osteotomy, but none contained a large sample [8, 10, 11, 15]. We compared the seven cases in our study with previously reported ones which were summarized in Table 3. Among different studies, various methods have been introduced to perform the single-stage procedure. There was no consensus on the order of TKA and corrective osteotomy, and fixation of the osteotomy.

**Table 3:** Previously reported results of single-stage TKA and osteotomy in patients with supracondylar deformity

Authors (PY)	*N*	Mean age (range), years	Deformity (type/*n*/degrees)	Mean follow-up (range), months	Which is first (TKA or osteotomy)	Type of implant	Fixation of osteotomy	Complications	Revision
Lonner et al. [10] (2000)	11	63 (40-74)	Varus/10/(14-40) Antecurvatum/7/(5-30) Internal rotation/1/45	46 (26-88)	Osteotomy	PS 11	Press-fit long stem 2 Plate 7 IM nail 2	Nonunion 1 (press-fit stem) Stiffness 2 Pulmonary embolism 1	0
Veltman et al. [8] (2015)	10	62 (43-82)	Varus/7/(6-22) Valgus/3/(11-17) Antecurvatum/1 Recurvatum/1 External rotation/3	(27-100)	Osteotomy	PS 11	K-wires/plate +short press-fit stem 10	Nonunion 2 Intraop fracture 1	1
Demir et al. [11] (2018)	10	63 (38-67)	Varus/9/(32-43) Antecurvatum/4/(27-35) Recurvatum/3/(23-33)	(31-60)	TKA	CR 9 UC 1	IM nail 9 IM nail+plate 1 (fracture)	Femoral component malposition 1 Intraop fracture 1 Deep infection 1	1
Catonné et al. [15] (2019)	6	64 (59-72)	Varus/3/(18-32) Valgus/2/(20-31) Recurvatum/1/15 Internal rotation/2/(10-40)	120 (60-180)	Osteotomy	PS 5 CCK 1	Press-fit stem (10-20 cm) 2 Press-fit stem (10-20 cm) +plate 4	Deep vein thrombosis 1 Stiffness 1	0
This study	7	62 (37-76)	Varus/2/(17-18) Valgus/4/(12-16) Antecurvatum/2/(34-39) Recurvatum/1/36	79 (38-104)	Osteotomy	PS 6 CCK 1	Long cemented stem (12.5-17.5 cm)	Fat liquefaction 1 Nonunion 1 Intraop fracture 1	0

*PY* publication year, *PS* posterior stabilized, *CR* cruciate retaining, *UC* ultra-congruent, *CCK* constrained condylar knee, *IM* intramedullary

Ideally, TKA and deformity correction can be combined successfully in a single setting without altering the individual methods of each. However, the problem is to prioritize which to perform first. Some authors thought it was more advantageous to correct the deformity after implanting the arthroplasty components to obtain the appropriate mechanical axis [11]. However, it was difficult to perform distal femoral resection using routine intramedullary alignment due to supracondylar deformity. On this occasion, computerized navigation will be helpful to obtain good alignment [16]. Conversely, authors supporting the “osteotomy first” technique thought that even if moderate deformity remained after osteotomy, mechanical axis could still be restored by using intra-articular bone cuts. Perhaps, to a larger extent, the order of TKA or osteotomy depends on the surgeon’s strengths and experience. In this study, we conventionally performed the osteotomy first. Then, we relied on the long intramedullary rod to orientate the femoral anatomical axis and engage the distal cortex after osteotomy, and further determine the appropriate resection angle. As a result, 90.79±2.40° of mLDFA value was acquired in our series, which showed the priority of using long extension stem. It had to be mentioned that although we did not use any temporary screws or k-wires to stabilize the osteotomy, sometimes the rotational control was inadequate solely by the intramedullary rod, which might need the aid of clips.

The options for fixation have expanded considerably to include long-stemmed prosthesis, retrograde intramedullary nails, k-wires, and locking distal femoral plates [2, 8, 10, 11, 15, 16]. Although plating system was the classic method of direct osteosynthesis, it was often associated with delayed bone healing due to the larger incision and greater disruption of the soft tissue envelope. Besides, on account of the need for partial weight bearing and increased contents around the knee joint, it would also compromise the recuperation of mobility [1]. By contrast, intramedullary nails could share load transfer and provide the advantage in the healing of the osteotomy line both mechanically and physiologically. In particular, the intramedullary lengthening nails might be appropriate for patients with post-traumatic limb length discrepancy [11, 17]. However, to permit retrograde nail insertion, the femoral component had to be an open-box design, which was usually inadequate for severely deformed knee [18]. The use of long press-fit stem was recommended by many authors [8, 10, 15, 19, 20]. By achieving optimal press-fit, it offered several benefits, such as simplicity and ease of application, rotational control of the osteotomy site, and the possibility of early weight bearing. However, the plate would be used finally if satisfactory stability were not achieved by using the long press-fit stem [15, 21].

In this study, we recommended the use of long cemented stem for osteotomy fixation which has been described by Papadopoulos et al. [22]. We supposed that long cemented stem could provide more reliable and instant stability than long press-fit stem. One big concern was the possibility of interposing cement into the osteotomy site, which caused one case of nonunion in our series. But we believed that it was a trap which can be avoided after practicing cementing technique [14]. Another concern was the potential revision procedure for the relative young patients, with the need of extracting the long cemented stem. There was also a split fracture of the distal femur during implanting the femoral component. Apart from excessive hammering force, we thought it was associated with the relatively lower osteotomy position which was close to the joint line. However, stems may be inappropriate in cases with deformity located far away from the joint line. Namely, the stem length had great influence on the stability. In our series, all cases had supracondylar deformity, instead of subtrochanteric deformity. With the longest stem up to 175 mm, the femoral canal isthmus was engaged in all cases, which warranted the stability of the osteotomy.

There are some limitations in our study. Firstly, due to the complexity and low incidence of these EADs, our study was limited by the small sample size. Secondly, this retrospective study did not give much detail of the osteotomy wedge. Thirdly, there was no available control group which applied other strategies including isolated TKAs, two-stage procedures, and any other fixation methods. However, we provided an atypical method with long-term follow-up, which supported the longevity of the components and validated the effectiveness of this method.

## Conclusions

For knee osteoarthritis with femoral supracondylar deformity, single-stage TKA and corrective osteotomy was feasible but technically demanding. The use of long cemented stem for osteotomy fixation can provide reliable rotational control of the bone segments. However, the sandwiched cement should be avoided to ensure bone healing.

## Data Availability

All data generated or analyzed during this study are included in this published article.

## Abbreviations

EAD: Extra-articular deformity
TKA: Total knee arthroplasty
HSS: Hospital of Special Surgery
ROM: Range of motion
MAD: Mechanical axis deviation
mLDFA: Mechanical lateral distal femoral angle
mMPTA: Mechanical proximal tibial angle
JLCA: Joint line congruence angle
